# Virtual Reality for Preprocedure Planning of Covered Stent Correction of Superior Sinus Venosus Atrial Septal Defects

**DOI:** 10.1161/CIRCINTERVENTIONS.123.013964

**Published:** 2024-11-05

**Authors:** Natasha Stephenson, Eric Rosenthal, Matthew Jones, Shujie Deng, Gavin Wheeler, Kuberan Pushparajah, Julia A. Schnabel, John M. Simpson

**Affiliations:** School of Biomedical Engineering and Imaging Sciences, King’s College London, United Kingdom (N.S., S.D., G.W., K.P., J.A.S., J.M.S.).; Department of Congenital Heart Disease, Evelina London Children’s Hospital, United Kingdom (N.S., E.R., M.J., K.P., J.M.S.).; School of Computation, Information and Technology, Technical University of Munich, Germany (J.A.S.).; Institute of Machine Learning in Biomedical Engineering, Helmholtz Munich, Germany (J.A.S.).

**Keywords:** catheterization, heart septal defects, atrial, pulmonary veins, virtual reality

## Abstract

**BACKGROUND:**

Covered stent correction (CSC) of a superior sinus venosus atrial septal defect is an alternative to surgery in selected patients, but anatomic variation means that assessment for CSC requires a 3-dimensional anatomic understanding. Heart VR is a virtual reality (VR) system that rapidly displays and renders multimodality imaging without prior image segmentation. The aim of this study was to evaluate the performance of the Heart VR system to assess patient suitability for CSC.

**METHODS:**

In a blinded fashion, 2 interventionalists reviewed preprocedural computed tomography scans using Heart VR to assess the feasibility of CSC, including the potential need for pulmonary vein protection. The total review time using VR was recorded.

**RESULTS:**

Using conventional imaging, 15 patients were deemed suitable for CSC, but at catheterization, 3 cases were unsuitable. Using VR, when both interventionalists agreed that a case was suitable for CSC (n=12), all proved technically feasible. In the 3 cases that were unsuitable for CSC, the interventionalists using VR were either uncertain (n=1) or did not agree on suitability (n=2). The strategy for pulmonary vein protection was correctly identified by interventionalist 1 and 2 in 9/12 and 8/12 cases, respectively. In cases where pulmonary vein protection was required intraprocedurally (n=5), this was correctly identified using Heart VR. Using VR, in 3 cases it was determined that pulmonary vein protection would be required, but this was not the case on balloon interrogation. VR data loading and review times were 82 seconds and 7 minutes, respectively. Verbal feedback indicated that Heart VR assisted in the assessment of case suitability.

**CONCLUSIONS:**

Heart VR is a rapid and effective tool for predicting suitability for CSC in patients with a superior sinus venosus atrial septal defect and could be a feasible alternative to segmented virtual or physical 3-dimensional models.

**WHAT IS KNOWN**

- Superior sinus venosus atrial septal defects can be successfully treated using covered stent correction instead of surgery.
- The wide anatomic variation necessitates preprocedure planning using 3-dimensional imaging to predetermine suitability.
- Virtual reality is a technology that can assist with 3-dimensional understanding of complex cardiac lesions.

**WHAT THE STUDY ADDS**

- Virtual reality was an effective and rapid tool for procedural planning of covered stent correction of sinus venosus atrial septal defect
- Virtual reality could offer a cheaper and quicker alternative to 3-dimensional printing for procedure planning in this lesion.

Superior sinus venosus atrial septal defect (SVASD) is characterized by a superior interatrial communication at the superior vena cava (SVC)–right atrial junction associated with anomalous drainage of ≥1 right-sided pulmonary veins (PVs).^1^ In recent years, covered stent correction (CSC) has emerged as an alternative to surgery in selected cases. The procedure involves the insertion of a covered stent into the SVC, which directs flow through the stent into the right atrium while the anomalous PV flow is directed behind the stent into the left atrium and abolishes the shunt.^2,3^ Over 50 patients have now been successfully treated at our center. Diagnosis and suitability for CSC are currently achieved by imaging techniques, including computed tomography (CT) and magnetic resonance imaging.^4^ These are interrogated on a flat screen either as multiplanar reformatted images or 3-dimensional (3D) volume-rendered or segmented reconstructions. 3D-printed models with physical stents have also been used to guide the optimal approach.^5,6^

Key considerations include secure placement of the covered stent in the SVC, flaring of the lower portion of the stent to avoid residual shunt, and avoidance of compression of the PVs redirected to the left atrium. The latter complication can often be avoided by simultaneous noncompliant balloon expansion in affected PVs to mold the SVC stent and increase the size of the new PV pathway (PV protection [PVP]).

The aim of this article is to report the use of a novel prototype virtual reality (VR) system (Heart VR) to investigate the potential use of this approach to assist the patient selection in a cohort of patients who had undergone attempted CSC for SVASD. This included the assessment of the feasibility of CSC and the requirement for PVP during the catheterization procedure.

## Methods

The data that support the findings of this study are available from the corresponding author upon reasonable request.

### Patient Selection and Procedural Details

Sixty-five patients underwent assessment for CSC at our center between January 2017 and December 2022. We used CT data for superior spatial resolution. Forty patients underwent CT of sufficient quality (<1 mm slice thickness, good right heart contrast enhancement, no significant motion artifact), 2 underwent suboptimal CT, and 23 underwent magnetic resonance imaging. Of the 40 selected patients, 26 underwent CSC, 5 underwent catheterization and balloon testing but did not proceed to CSC, and 9 were referred directly for surgery. Patients referred directly for surgery were excluded, leaving 31 eligible patients. Fifteen patients were randomly selected from this cohort for the study.

All included cases were deemed suitable for CSC using standard imaging, which included CT review using cross-sectional multiplanar reformatted on Sectra PACS (Sectra AB, Linkoping, Sweden) and as a segmented volume-rendered 3D model on Syngo.via (Siemens Healthineers, Erlangen, Germany), displayed on a 2-dimensional (2D) screen meeting minimum standards for radiology use.^7^ Patient-specific 3D-printed models were created in 2 cases to clarify anatomy. Preprocedure documentation of the need for PVP using these techniques was not recorded. For the purpose of this study, the views of the interventionalists before the procedure were used, and the standard imaging was not reinterrogated.

Cardiac catheterization and CSC were performed as previously described by Hansen et al.^3^

### Imaging Data

Contrast-enhanced CT scans from our center and external referral centers were used. At our center, prospective electrocardiogram-gated contrast-enhanced CT angiography was performed on a Siemens Dual Source Flash multidetector scanner during inspiratory breath hold with 0.6-mm slice thickness. External scans had variable acquisition protocols, but the minimum acceptable spatial resolution for study inclusion was 1 mm, with good right heart contrast opacification and a field of view that included the SVC to the insertion of the innominate vein and the right-sided PVs.

### VR Visualization

The Heart VR system was created in-house via Unity (a video game development platform; Unity Technologies, San Francisco, CA) with the inclusion of Insight Toolkit (a visualization library specifically designed for scientific imaging), using a plugin system to enable the loading of imaging data.^8^ For VR visualization, digital imaging and communication in medicine files of CT studies were loaded directly into the VR system and displayed as volume-rendered objects in the virtual scene (Figure S1). No presegmentation of data was required: external and internal cardiac and vascular surfaces could be automatically rendered via real-time adjustment of windowing and transfer functions. VR software was displayed and interacted with via an HP Reverb G2 headset and controllers (Hewlett Packard Company, Palo Alto, CA). The CT volume could be moved, rotated, cropped, and measured using an in-built tool panel. Virtual stent models could be placed in the scene, and the size adjusted using the handheld controllers. The time taken to load each data set was recorded.

### Digital Stent Modeling

**Figure 1.**
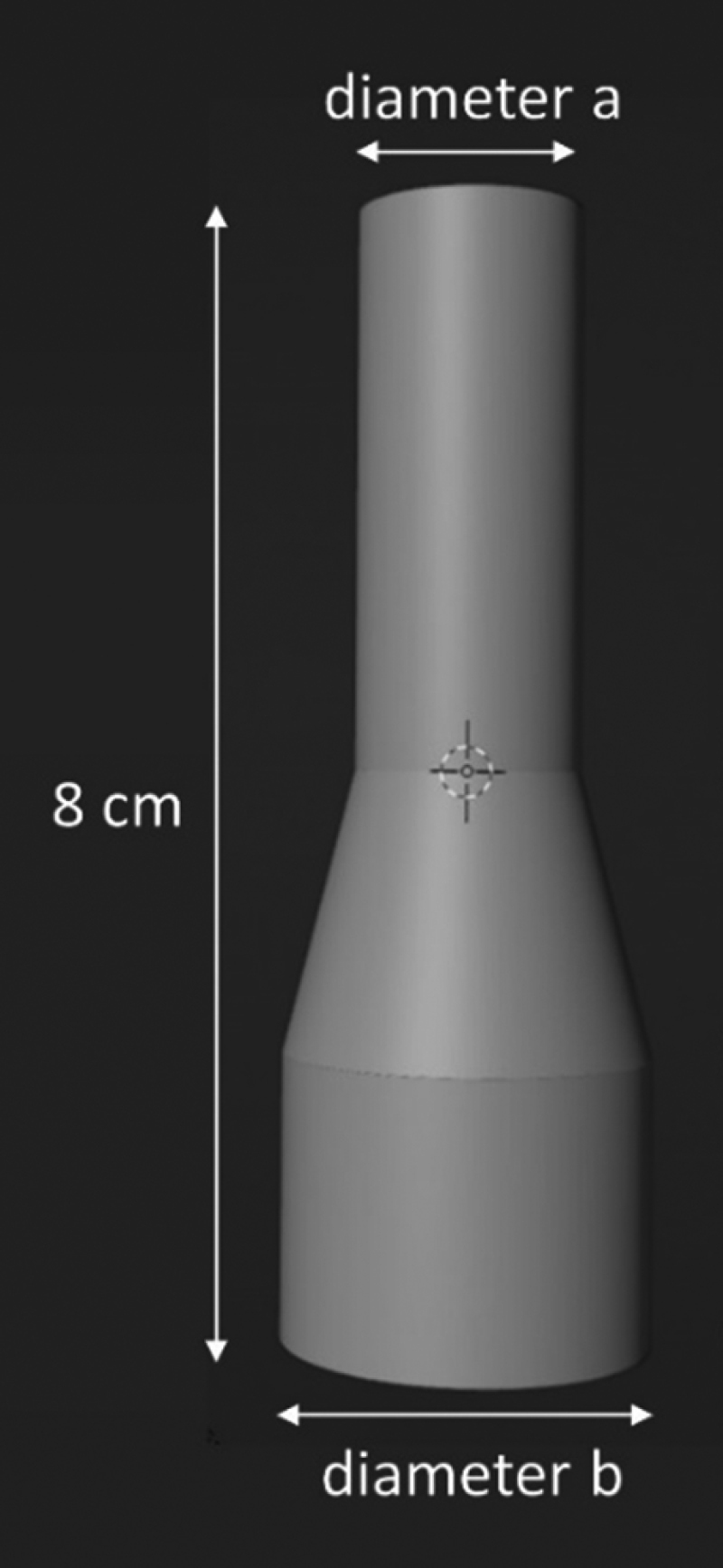
Virtual stent with labeled diameters.

Virtual stents placed within the VR visualization were generated using Blender software (version 3.3.1; Blender Foundation, Amsterdam).^9^ All stents were 80 mm in length with a 40-mm upper cylinder, a 20-mm truncated cone, and a 20-mm lower cylinder (Figure 1). The diameter of the upper cylinder (a) and lower cylinder (b) were adjusted in 2-mm increments (diameter a, 10–22 mm; diameter b, 24–30 mm), producing a total of 28 stent models. The meshes were solidified and exported as Standard Tessellation Language files to enable incorporation into VR.

### Study Protocol

**Figure 2.**
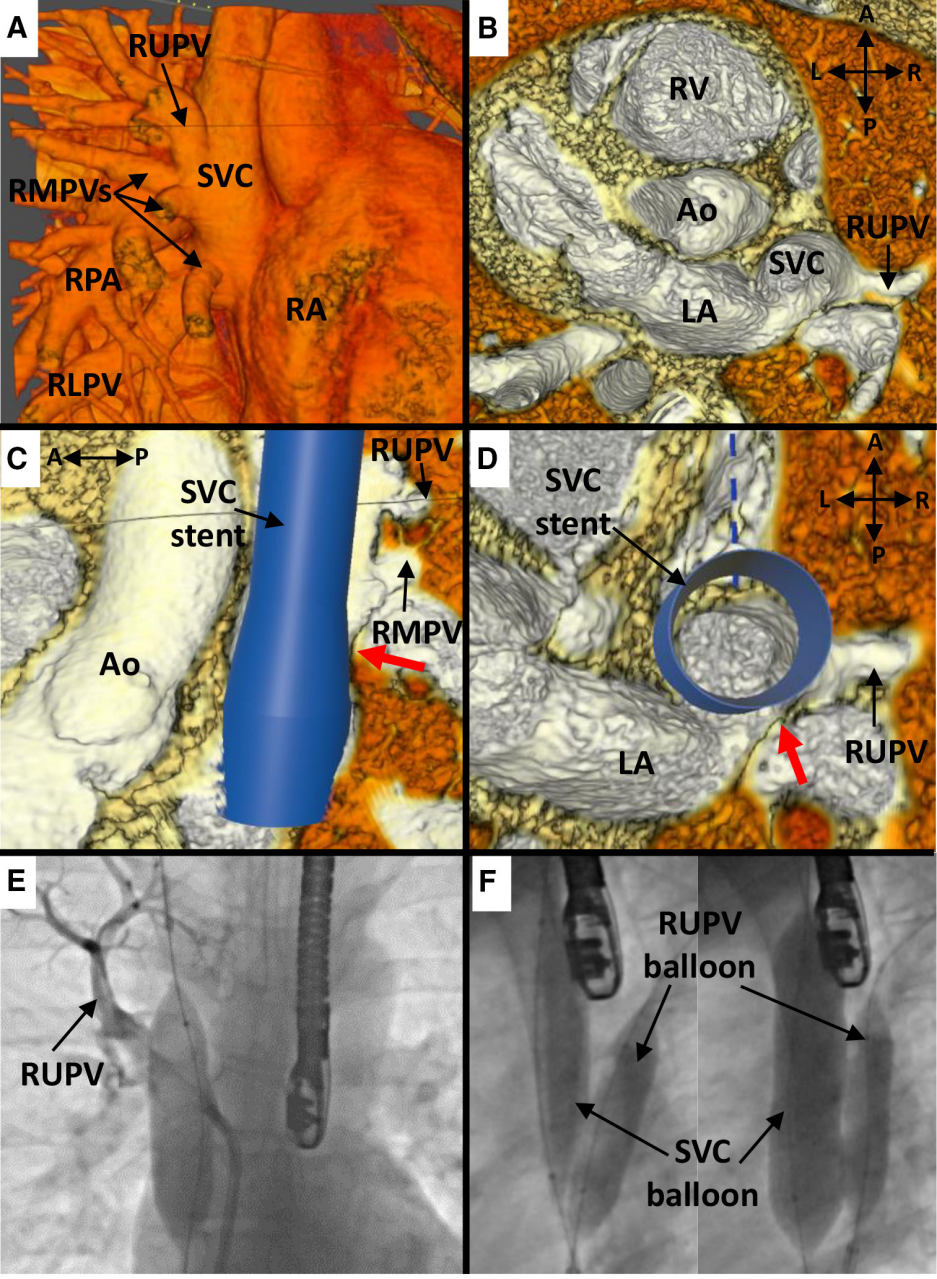
**Case 9. A**, Anterior view of superior vena cava (SVC) and pulmonary vein (PV) anatomy in virtual reality (VR), showing entry of a large right upper PV (RUPV) and right middle PVs (RMPVs) into the SVC. **B**, Superior view: internal view showing the entry point of the RUPV to the right and superior to the interatrial communication. **C**, VR internal view from the left showing the point of potential obstruction of the PV pathway (red arrow) after stent insertion. **D**, Superior view with virtual stent in situ showing a narrow pathway (red arrow) of RUPV through to the left atrium (LA) and potential for obstruction. **E**, Angiogram in anteroposterior (AP) projection with 20-mm AltoSa-XL balloon inflated in the SVC with simultaneous RUPV angiography showing hold-up of contrast in the RUPV, signifying obstruction. **F**, Angiogram from left anterior oblique (LAO) projection showing (L) a partially inflated AltoSa-XL balloon in the SVC and simultaneous Atlas Gold 12×4 mm balloon in the RUPV, and (R) after full inflation of the SVC balloon showing significant posterior displacement of the RUPV balloon, signifying that PV obstruction would likely occur despite balloon protection. Ao indicates aorta; RA, right atrium; and RV, right ventricle.

Ethical approval was obtained from the National Health Service Health Research Authority for the use of pseudoanonymized patient imaging data for retrospective studies (Research Ethics Committee reference: 21/LO/0650). Given the conditions of data use, individual patient consent was not required within the institutional review board. Two interventional cardiologists (interventionalist 1 and interventionalist 2) were selected to participate in the study. Interventionalist 1 has performed/supervised over 50 CSC cases, and interventionalist 2 has performed/supervised 40 CSC cases. Both interventionalists were given a 15-minute orientation to the VR system and analyzed 1 SVASD data set before commencing the study. All 15 cases were anonymized and presented in random order. The interventionalists were asked to determine whether the cases were suitable or unsuitable for CSC using Heart VR (Figure 2). Where an interventionalist could not reach a definitive conclusion regarding suitability based on the VR visualization, it was classified as uncertain. If the case was determined to be suitable, the interventionalist was also asked to assess the need for PVP based on VR review. Results of the VR review were compared with the outcomes from invasive cardiac catheterization. Total VR data load time (to the nearest second) and the total time for data review and decision-making (to the nearest minute) were recorded and expressed as median values±interquartile range.

After completion of all VR cases, both interventionalists completed a questionnaire about the VR system: usability, image quality, ease of stent placement/adjustment, added value, confidence for case selection compared with 2D techniques and 3D prints, and time-saving value (Supplemental Methods). Free-text comments could also be recorded.

### Statistical Analysis

Given the retrospective nature of the study with 15 cases, results are presented descriptively rather than by statistical methods.

## Results

### Study Population

CSC was attempted in all 15 patients (Table S1). The median age was 56.6 years (range, 29.6–73.9), and 6/15 (40%) were women. In all cases, a patient-specific volume-rendered 3D model displayed on a 2D screen interface had been used to assist in preprocedure planning. In 2 cases, 3D-printed models were also used to assess suitability. Preprocedure comments regarding PVP using these imaging techniques had not been documented.

CSC was performed in 12 patients (80%), of whom 5 had intraprocedural PVP (Table S2). Postprocedure transesophageal echocardiography assessment confirmed no residual shunt in 4 patients, trivial shunts in 2, and a mild residual shunt in 6. No patients had evidence of PV stenosis.

In 3 cases, CSC was not performed following balloon interrogation. In 2 patients, this was due to angiographic evidence of PV compression that did not appear amenable to protection (cases 9 and 10). In the third case (patient 7), at least 2 large right upper PV branches entering high into the SVC were not divertible with stenting, with evidence of compression of the right middle PV branch on balloon testing.

### Assessment of Suitability Using VR

In all cases where CSC was successful (n=12), both interventionalists correctly assessed them as suitable using VR (Table S2). In cases that were determined unsuitable at the time of catheterization (n=3), interventionalists 1 and 2 were each correct in 1 case, uncertain in 1, and incorrect in 1. In all unsuitable cases, at least 1 of the interventionalists either disagreed on feasibility or expressed uncertainty.

In case 9, where balloon testing showed PV occlusion not amenable to PVP at catheterization, both interventionalists were uncertain of suitability based on VR review (Figure 2). In their verbal comments, both expressed concerns regarding the adequacy of the PV pathway around an implanted stent but suggested that they would consider offering cardiac catheterization to determine suitability.

**Figure 3.**
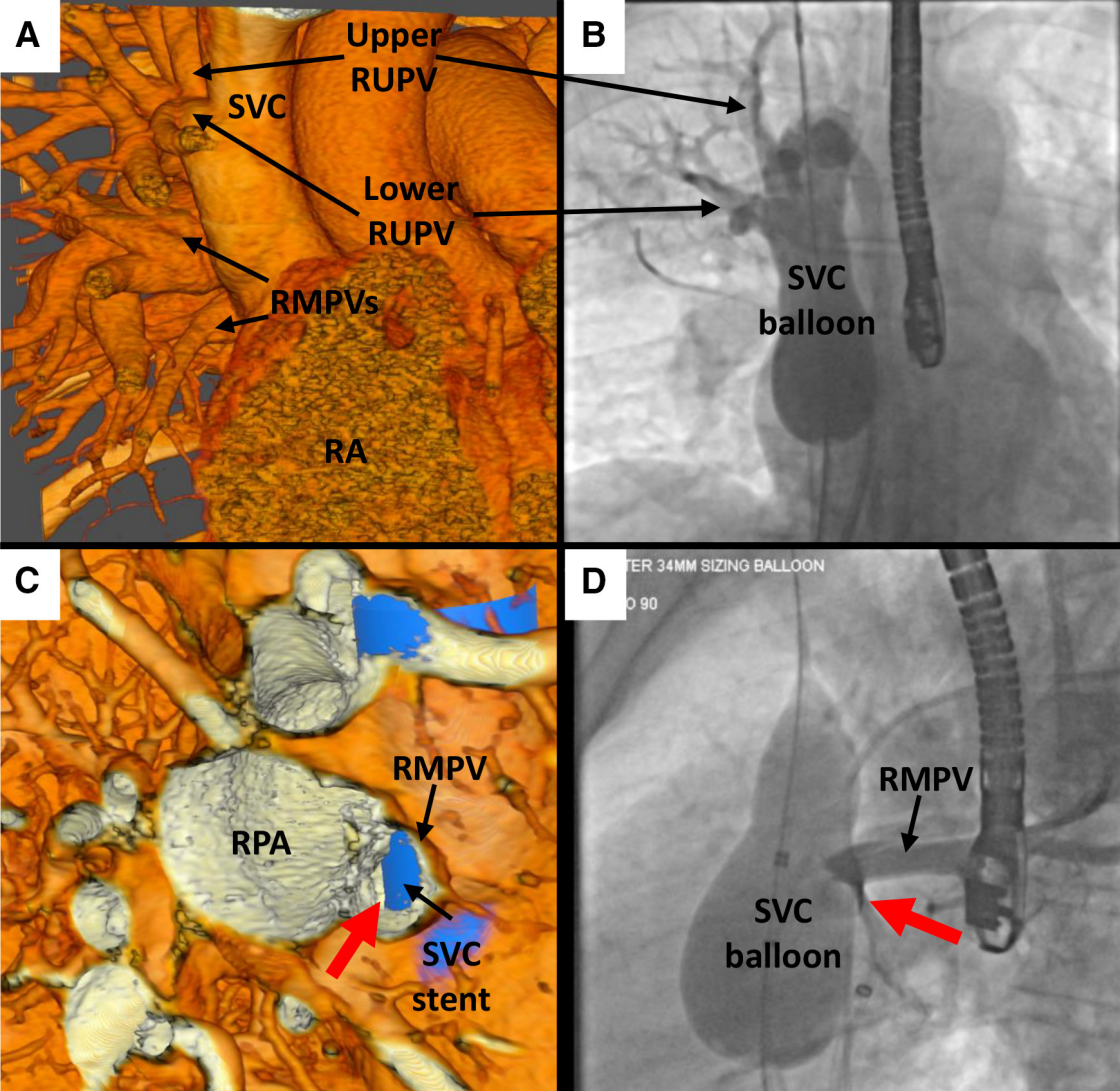
**Case 7. A**, Anterior external view in virtual reality (VR) showing analogous pulmonary vein (PV) anatomy demonstrated on angiography in **B**, with a large upper right upper PV (RUPV) entering high in the superior vena cava (SVC) and a separate lower RUPV entering just below, and both veins entering too superior to be diverted to the left atrium (LA) after stenting. **C**, VR view from the right toward the orifice of the right middle PV (RMPV) into the SVC with a virtual SVC stent in situ there is potential for at least partial obstruction of the RMPV, with a small pathway (red arrow) posterior to the stent to the LA. **D**, Angiographic image in the left anterior oblique (LAO) view showing hold-up of contrast in the RMPV after selective angiography with SVC balloon inflation, with the small PV pathway (red arrow) seen. RA indicates right atrium; and RPA, right pulmonary artery.

In case 7, there was evidence of obstruction of right middle PV flow during balloon testing, visualizable in VR and correctly identified by interventionalist 1 (Figure 3). Both interventionalists noted the high anomalous PV; however, interventionalist 1 felt it was small and could be left draining to the SVC, whereas interventionalist 2 identified that this was a large nondivertible vein.

**Figure 4.**
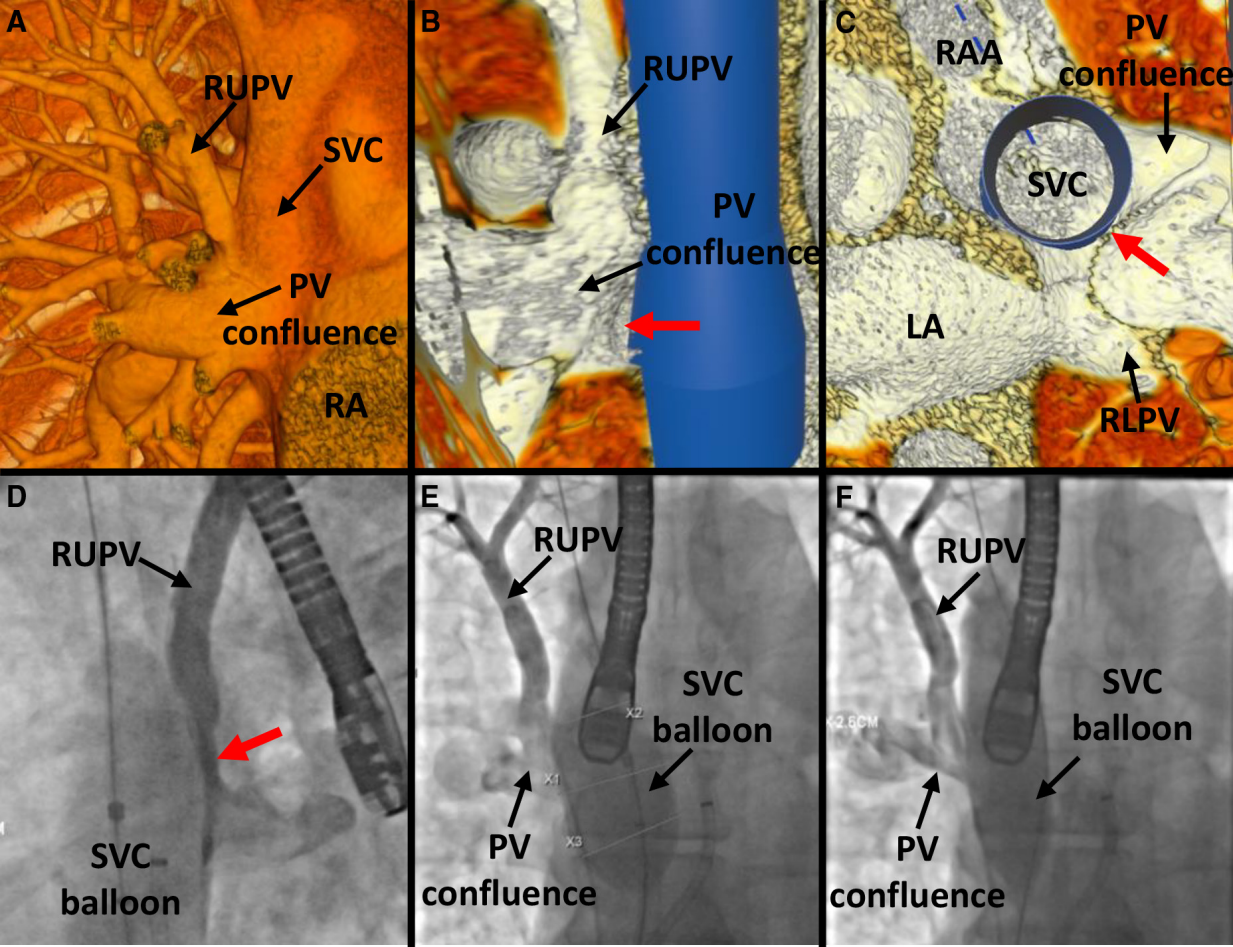
**Case 10. A**, Anterior view in virtual reality (VR) showing a separate right upper pulmonary vein (RUPV) entering a larger pulmonary vein (PV) confluence in the superior vena cava–right atrial (SVC-RA) junction. **B**, Internal view in VR from the right showing the PV confluence entering into the SVC-RA junction with a virtual stent in situ, resulting in a narrow PV pathway through to the left atrium (LA; red arrow). **C**, Superior view in VR with the SVC stent in situ, showing a narrow PV pathway through to the LA. **D**, Left anterior oblique and (**E**) anteroposterior (AP) angiographic projections after right PV injection, showing hold-up of contrast in the RUPV and the small PV pathway to the LA (red arrow) after 34 mm balloon inflation in the SVC. **F**, AP projection after right PV injection showing ongoing obstruction despite downsizing the SVC balloon to 26 mm. RA indicates right atrium; RAA, right atrial appendage; and RLPV, right lower pulmonary vein.

In case 10, interventionalist 1 correctly identified that the right upper PV would be subject to obstruction if a stent was placed in the SVC, whereas interventionalist 2 felt CSC might be possible with PVP (Figure 4).

### Assessment for PVP

**Figure 5.**
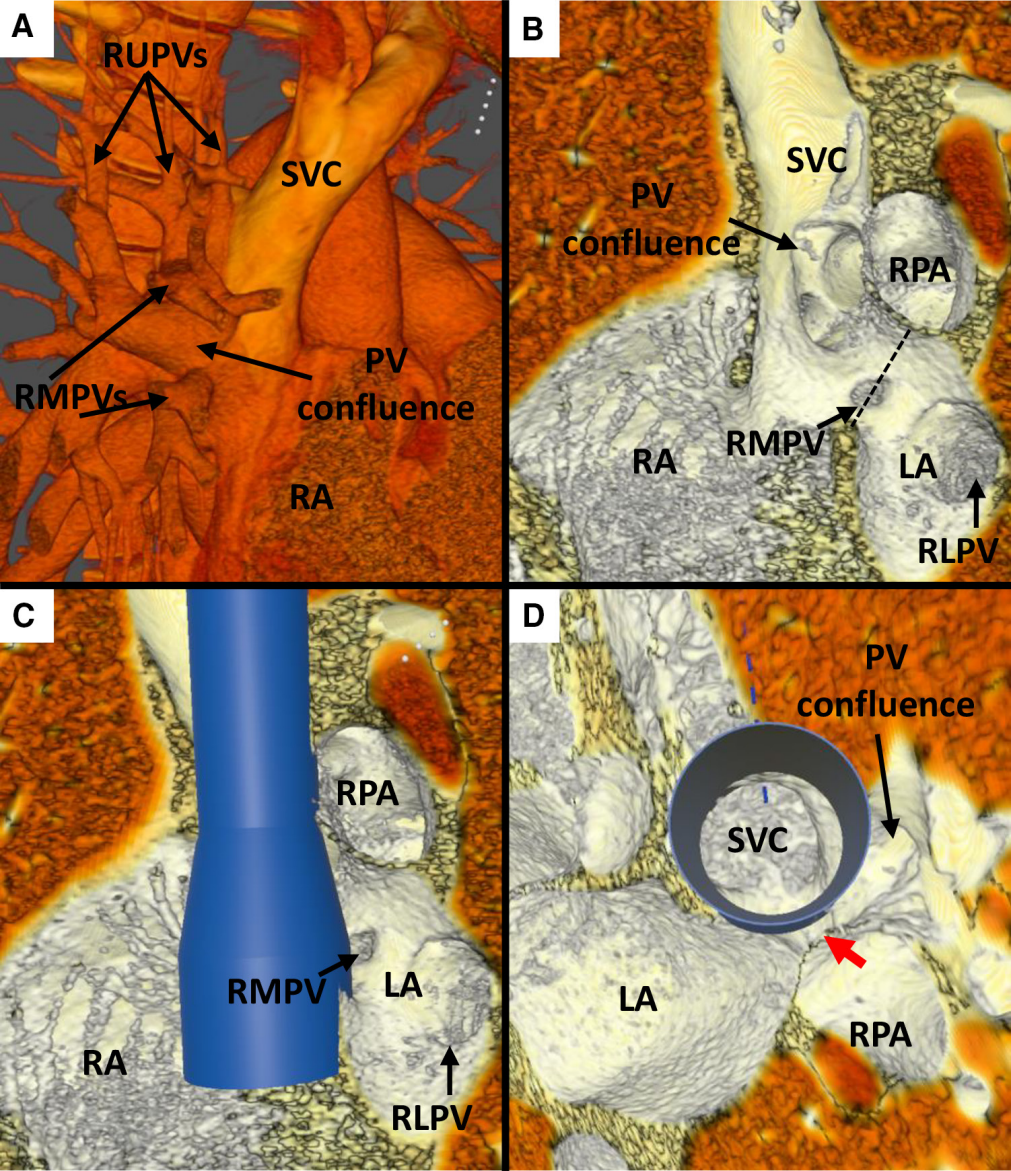
**Case 1. A**, Multiple right upper pulmonary veins (RUPVs) and right middle pulmonary veins (RMPVs) forming a large confluence in the superior vena cava (SVC). **B**, Left sagittal view: internal anatomy of the SVC–right atrial junction, with multiple RUPVs and RMPVs forming the confluence and an additional small RMPV entering at the level of the sinus venosus defect (dashed line) and right lower pulmonary vein (RLPV) connecting normally to the left atrium (LA). **C**, Virtual stent in situ: the pulmonary vein (PV) confluence would need to drain posteriorly around the stent to enter the LA. **D**, Superior view showing the virtual stent in the SVC at the level of the PV confluence, with a relatively narrow pathway (red arrow) for drainage of the multiple anomalous PVs. RA indicates right atrium; and RPA, right pulmonary artery.

Using VR, the PVP strategy used intraprocedurally was correctly identified by interventionalist 1 in 9 of 12 cases and by interventionalist 2 in 8 of 12 cases (Table S2). PVP was performed in 5 of 12 cases. For all 5 cases where PVP was performed, this was correctly identified by both interventionalists using VR (Figure 5). In cases where PVP was not performed, the interventionalists could identify this using VR in the majority of cases. Interventionalist 1 had 3 and interventionalist 2 had 4 false-positive assessments where using VR determined that PVP would be required, when this was not the case on diagnostic catheterization. No false-negative assessments regarding PVP were recorded.

### Review Time

The median review time in VR for interventionalist 1 was 12 (interquartile range, 8) minutes and 5 (interquartile range, 3) minutes for interventionalist 2. The median total review time for both interventionalists was 7 minutes. The median time for data loading in VR was 82 (interquartile range, 32.8) seconds.

### Questionnaire Results

The Likert scale questionnaire results are summarized in the Table, where 1 equates to the most negative response and 5 to the most positive.

**Table.**
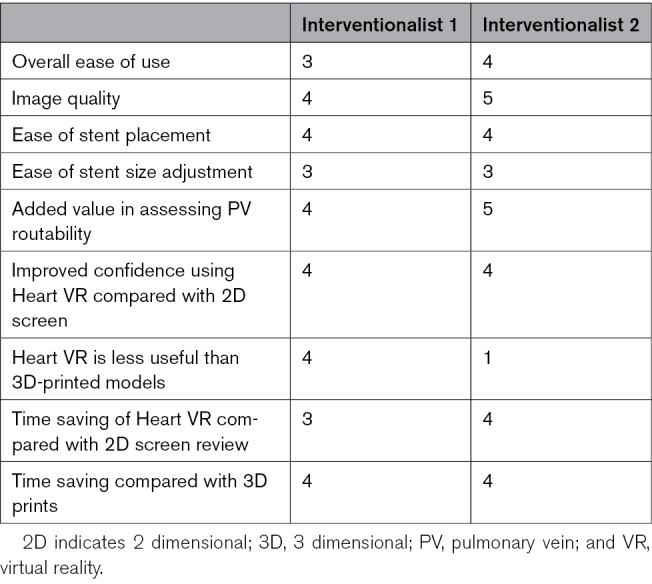
Questionnaire Responses on Use of Virtual Reality for Covered Stent Correction of Sinus Venosus Defects

In free-text comments, it was noted that VR can give additional clarity about the procedure compared with using multiplanar reformatted images or volume-rendered 3D reconstructions displayed on a flat screen. The ability to place stents within the VR model made it superior. Being able to visualize the whole volume of data in VR was felt to be beneficial and improved confidence in case selection compared with display on a 2D screen. However, they felt that user interaction for stent placement and adjustment could be improved. One interventionalist reported that they found 3D prints easier to use than VR, as holding a 3D print felt less cumbersome than VR; however, the other found VR easy to use following a shallow learning curve.

## Discussion

SVASDs are innately 3D lesions and highly heterogeneous between patients.^4^ Interrogation of echocardiograms and cross-sectional imaging on a flat screen using multiplanar reformatted slices or 3D-rendered images is currently the standard of care. However, when assessing SVASDs for transcatheter closure, these methods are imperfect. When transesophageal echocardiography was used for patient selection, 40% of selected patients were found to be unsuitable for CSC due to PV obstruction at catheterization.^10^ Review of CT and magnetic resonance imaging using multiplanar reformatted images on a flat screen has become the dominant imaging approach for case selection in CSC. However, this does not typically permit the insertion of virtual devices to simulate drainage of anomalous PVs following stent placement. Printed 3D models have provided an alternative means of assessment, allowing implantation of real stents to simulate the procedure. However, 3D printing is not universally available, involves additional time and costs to segment images print models, and wastes real devices such as stents within the models.

Our article demonstrates that the use of a VR system for patient selection in SVASD is feasible. All 15 cases were successfully loaded, visualized, and interrogated in the virtual environments directly from digital imaging and communication in medicine images. In addition, virtual adjustable stents were easily placed and manipulated within the VR environment. Both interventionalists were correct in their VR assessment of suitability in 13 of 15 cases, uncertain in 1 of 15, and incorrect in 1 of 15. This compared with the correct assessment being made in 12 of 15 cases using conventional imaging pre-procedure. Using VR, where both interventionalists agreed on suitability for CSC, there was procedural success in all cases. Interestingly, where interventionalists disagreed (n=2) or were uncertain (n=1) about suitability, CSC was not feasible at catheterization.

The correct strategy for PVP was identified by interventionalist 1 in 9 of 12 cases (75%) and by interventionalist 2 in 8 of 12 cases (67%) using VR. There were 3 cases in which interventionalist 1 and 4 cases in which interventionalist 2 incorrectly assessed that they would perform PVP using VR when this was not required during catheterization. However, in all 5 cases where PVP was required intraprocedurally, this was always correctly identified in VR. Although there was no documented preprocedural prediction of PVP using standard imaging for comparison with VR, these results suggest that VR assessment may be a beneficial tool for predicting procedure modifications.

Both interventionalists reported that stent placement and adjustment in VR assisted their assessment of the various defects, enabling a rapid and more confident understanding of patient suitability for the procedure, especially in cases where 2D imaging is unclear. VR enables both realistic depth perception and intuitive interaction with virtual objects in 3D space through the use of a stereoscopic headset and hand-held controllers, which may provide a better understanding of the data compared with desktop modalities. Case reports have shown that VR can be used to plan CSC.^11,12^ Tandon et al^13^ incorporated cylindrical stents into VR segmentations from 28 patients with SVASD, determining that 40% of patients would be suitable for CSC and a further 25% would be equivocal due to possible obstruction of a small PV by the stent. They proposed that VR visualization could assist with patient selection in SVASD; however, patient outcomes were not reported and as such this conclusion cannot be validated. In this study, by comparing the review of imaging in Heart VR to catheter outcome data and standard imaging review, we have shown promising initial results for VR to potentially enhance procedure planning in CSC for SVASD.

Comments from study participants have highlighted key benefits of VR in procedure planning. Both interventionalists commented that the rapidity of image loading is a benefit of Heart VR compared with other 3D modalities, the majority of which require image segmentation, which takes 30 to 120 minutes depending on complexity.^14^ The process of producing 3D prints is particularly time- and labor-intensive, necessitating an additional 35 hours for model printing and postprocessing on top of segmentation time.^15^ Most virtual modeling platforms, including commercially available VR programs, require segmentation either before image loading or within the VR environment. In contrast, Heart VR uses volume-rendering algorithms to load images directly from digital imaging and communication in medicine files and can produce solid or hollow models for interrogation in a median of 82 seconds. VR-embedded tools such as volume cropping also circumvent the need to destroy 3D-printed models to visualize internal structures. However, comments have also highlighted that improvements in the Heart VR system are still required to improve user-friendliness, including making stent placement and adjustment easier.

SVASD was selected as a study group for validation of VR imaging because of the complex and highly variable nature of the lesion and the pivotal nature of preprocedural imaging in appropriate case selection.^9^ Due to the retrospective nature and size of this study, we did not set out to demonstrate patient benefit or superiority of VR. However, the results suggest that VR is not inferior to conventional imaging for case selection in CSC of SVASD and has the benefit of being a rapid way of visualizing and interacting with complex 3D imaging data. Heart VR and other extended reality platforms have the potential to enable the creation of bespoke devices for patients who might otherwise be unsuitable for CSC, further broadening the reach of this procedure.^16^ Other groups have also used similar technologies for procedures such as transcatheter valve implantations^17^ or in more complex structural interventions such as stenting of systemic venous baffle obstruction post-atrial switch procedure.^18^

### Study Limitations

In 3 cases, following Heart VR review, both interventionalists had concerns regarding PV patency, which were not borne out intraprocedurally. In centers where PVP is not routine this might have led to these patients being incorrectly deemed unsuitable for CSC. Data suggest that in experienced centers, two-thirds of patients with superior SVASD are suitable to undergo CSC;^9^ however, previous studies using 3D imaging to predict suitability have underestimated this proportion.^4,13^ When Heart VR suggests the possibility of PV narrowing, it might improve the preparedness of the interventionalist to adopt a PVP strategy during catheterization, but it would not necessarily rule out successful CSC. A recent article with a larger experience showed that the feasibility of CSC for SVASD improved from 68% to 94% when a PVP strategy was used.^19^ In addition, most imaging techniques do not take into account factors such as tissue distensibility, which might affect the procedure outcome, and this remains a limitation of these techniques.

This study focused specifically on whether VR could assist with the assessment of PV anatomy; however, interventionalists were not specifically asked about residual shunt at the caudal end of the stent, another major factor determining procedural success in CSC. The VR stent models in this experiment were of standard length, and being unable to adjust this would make the assessment of residual shunt difficult. Future advances in VR stents and devices, including the ability to model for stent shortening and flare, may further improve accuracy.

The retrospective design of this study was selected because performing data collection prospectively was prohibited due to pending ethical and regulatory approvals of Heart VR, potentially leading to selection bias. The small sample size does not allow conclusions to be drawn regarding the superiority or inferiority of VR in this context. A prospective study with a larger patient cohort would be required to definitively determine the superiority or inferiority of VR to conventional imaging. To our knowledge, such a study has not been performed on any extended reality platform for cardiac structural intervention.^20^

### Conclusions

Detailed preprocedure assessment has been recognized as a key factor in achieving a successful outcome in CSC of SVASD. In this study, we have demonstrated that the Heart VR software enables accurate and reliable preprocedure assessment. In addition, the rapid loading of data directly from digital imaging and communication in medicine files enables imaging review and decision-making in a matter of minutes. From our results, VR could be a valuable imaging tool for stratifying patient suitability for CSC and assessing procedure modifications, with the potential to reduce unnecessary procedures and save time.

## ARTICLE INFORMATION

### Sources of Funding

This study was supported by the British Heart Foundation (BHF) funded 3D Heart Project (TA/F/20/210021) and the Evelina London Children’s Charity. It was also supported by previous funding from the National Institute for Health Research (NIHR) i4i grant (II-LA-0716–20001). The research was funded/supported by the NIHR Biomedical Research Center based at Guy’s and St Thomas’ NHS Foundation Trust and King’s College London and supported by the NIHR Clinical Research Facility at Guy’s and St Thomas’. The views expressed are those of the authors and do not necessarily reflect those of the National Health Service, BHF, NIHR, or the Department of Health.

### Disclosures

J.M. Simpson, Dr Schnabel, and K. Pushparajah are founder directors of XRnostics, a spinout company of King’s College London and Guy’s and St Thomas Hospitals Trust, which focuses on the use of extended reality imaging to assist in planning cardiac procedures. The collection and analysis of the data in this article predates the spin-off of the company. The other authors report no conflicts.

### Supplemental Material

Supplemental Methods

Tables S1–S2

Figure S1

## Supplementary Material


