# Supplementary material for: Virtual Reality for Preprocedure Planning of Covered Stent Correction of Superior Sinus Venosus Atrial Septal Defects
Source: Circ Cardiovasc Interv. 2024 Nov 5;17(12):e013964. doi: 10.1161/CIRCINTERVENTIONS.123.013964 (PMC7616809; doi:10.1161/CIRCINTERVENTIONS.123.013964)
Supplement: Supplementary file 1 [file hcv-17-e013964-s001.pdf]

SUPPLEMENTAL MATERIAL

Supplemental Methods: interventionalist questionnaire

1. Please rate the ease of use of the VR system as a whole

Very difficult	Difficult	OK	Easy	Very easy
1	2	3	4	5

2. Please rate image quality in VR

Very poor	Poor	Fair	Good	Very Good
1	2	3	4	5

3. Please rate ease of virtual stent placement in VR

Very difficult	Difficult	OK	Easy	Very easy
1	2	3	4	5

4. Please rate adjustment of device size in VR

Very difficult	Difficult	OK	Easy	Very easy
1	2	3	4	5

5. Heart VR has added value in assessing pulmonary vein routability compared to CT imaging viewed on a 2D screen

Strongly disagree	Disagree	Neither agree nor disagree	Agree	Strongly agree
1	2	3	4	5

6. a) Imaging review Heart VR improves my confidence in case selection for TCC in SVASD compared to CT review on a 2D screen.

Strongly disagree	Disagree	Neither agree nor disagree	Agree	Strongly agree
1	2	3	4	5

b) If you agree, please describe why?

7. a) Heart VR is less useful for procedure planning in TCC than 3D-printed models.

Strongly disagree	Disagree	Neither agree nor disagree	Agree	Strongly agree
1	2	3	4	5

b) Why?

8. Heart VR would save time in procedure planning for TCC compared to CT review on a 2D screen

Strongly disagree	Disagree	Neither agree nor disagree	Agree	Strongly agree
1	2	3	4	5

9. Heart VR would save time in procedure planning for TCC compared to 3D-printed models

Strongly disagree	Disagree	Neither agree nor disagree	Agree	Strongly agree
1	2	3	4	5

10. In your opinion, what is/are the most important feature(s) of Heart VR for procedure planning in SVASD?

Supplemental Figures

Figure S1: VR review in a CSC case.

A: anterior view of external anatomy showing anomalous drainage of right upper PVs (RUPVs) and one right middle PV (RMPV) into a confluence before entering the SVC. A single RMPV can be seen entering the superior vena cava/right atrial (SVC/RA) junction separately; B: anterior view showing internal anatomy of SVC-RA junction with the PV confluence draining into the superior vena cava (SVC) with relative location of the sinus venosus defect (SVASD, dashed line); C: sagittal cut through the SVASD viewed from the left showing the PV confluence entering the SVC, a small RUPV draining through a separate orifice more superiorly with the right lower vein (RLPV) entering the left atrium (LA); D: left posterior view cropped at level of SVASD, showing the virtual stent in position in the SVC, the inferior border of the SVASD (dashed line) is effectively occluded by the stent and a pathway for the anomalous PVs to drain posteriorly around the stent to the LA (red arrow); E: view from patient's right side showing stent positioned in the SVC and highlighting the posterior and superior borders of the SVASD (dashed line) through which anomalous PVs can drain to the LA (red arrow); F: superior view looking down at the stent in the SVC/RA junction and relative position of the PV confluence. There is a clear pathway (red arrow) for these veins to drain to the LA.

*Ao – aorta, RA – right atrium, RMPV – right middle pulmonary vein, RPA – right pulmonary artery

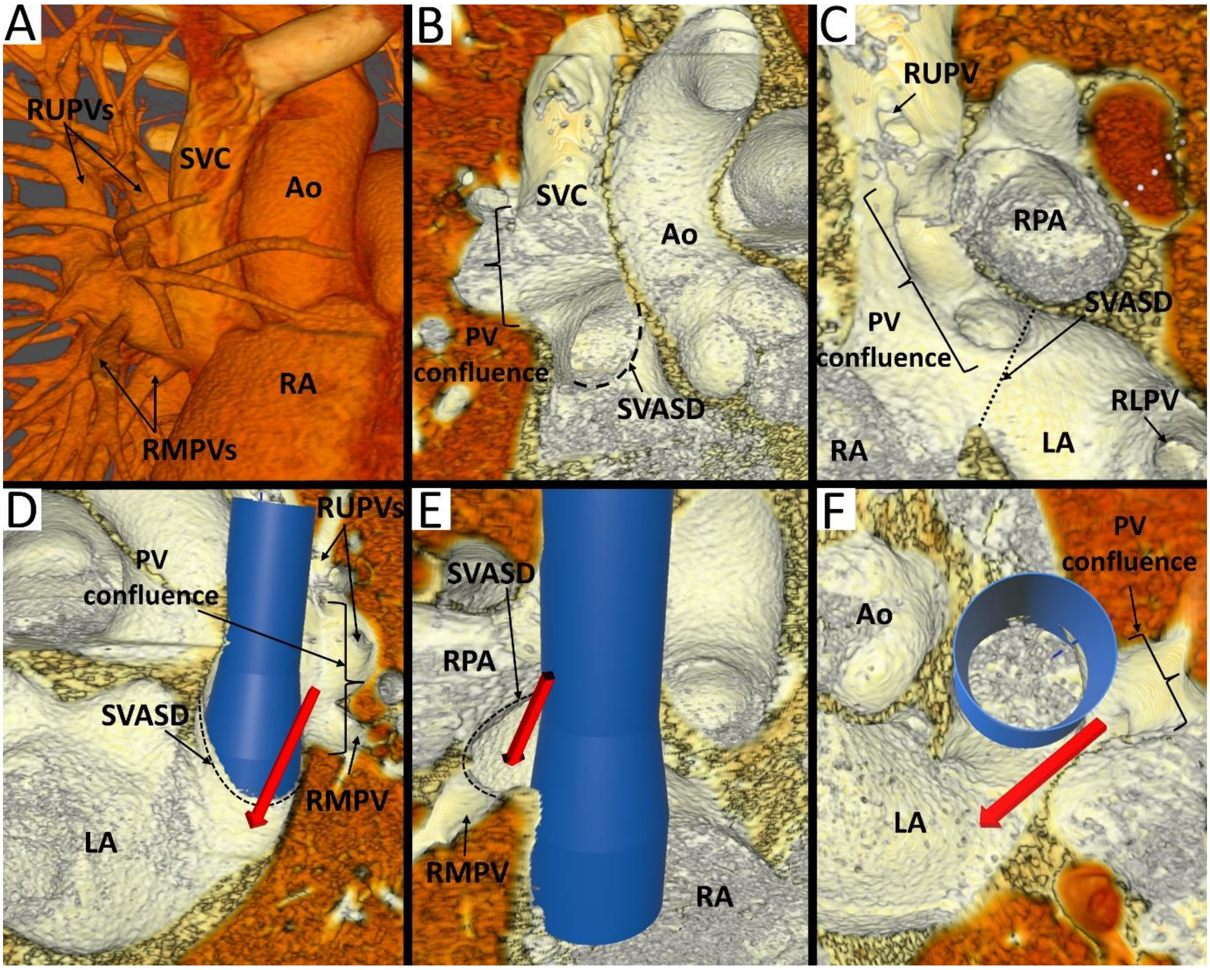
A
RUPVs
SVC
Ao
RA
RMPVs
B
SVC
Ao
PV
confluence
SVASD
C
RUPV
RPA
PV
confluence
SVASD
RLPV
RA
LA
D
PV
confluence
RUPVs
SVASD
RMPV
LA
E
SVASD
RPA
RMPV
RA
F
PV
confluence
Ao
LA

Supplemental Tables

Table S1: Patient pre- and post-procedure characteristics.

F – female; M – male; N – no; nd – no data; Y – yes

**Demographics**			**Pre-procedure imaging**		**Procedure**		**Post-procedure imaging**		
Case	Age (years)	Sex	3D model on 2D screen	3D print	CSC performed?	PVP?	PV obstruction post-procedure?	TOE residual shunt (grade)	MRI residual shunt (Qp:Qs)
**1**	30.2	F	Y	Y	Y	Y	N	trivial	1:1
**2**	59.0	M	Y	Y	Y	Y	N	mild	1.4:1
**3**	32.7	M	Y	N	Y	N	N	mild	nd
**4**	61.2	M	Y	N	Y	N	N	mild	1:1
**5**	46.1	F	Y	N	Y	N	N	mild	nd
**6**	64.4	M	Y	N	Y	N	N	none	1.1:1
**7**	64.9	F	Y	N	N	-	-	-	-
**8**	47.4	F	Y	N	Y	N	N	none	1:1
**9**	29.6	F	Y	N	N	-	-	-	-
**10**	53.3	M	Y	N	N	-	-	-	-
**11**	43.4	M	Y	N	Y	Y	N	trivial	1:1

**12**	67.1	M	Y	N	Y	Y	N	mild	nd
**13**	73.9	F	Y	N	Y	N	N	mild	1.8:1
**14**	56.6	M	Y	N	Y	N	N	none	nd
**15**	57.0	M	Y	N	Y	Y	N	none	nd

Table S2: VR assessments compared with catheter outcome for CSC suitability and PVP.

**Case**	**Pre-procedure imaging assessment**	**Catheter outcome**		**VR assessment: suitability for CSC**		**VR assessment: PVP**	
	**Suitable?**	**CSC success?**	**PVP?**	**Int 1**	**Int 2**	**Int 1**	**Int 2**
1	Y, correct	Y	Y	Y, correct	Y, correct	Y, correct	Y, correct
2	Y, correct	Y	Y	Y, correct	Y, correct	Y, correct	Y, correct
3	Y, correct	Y	N	Y, correct	Y, correct	*Y, incorrect*	*Y, incorrect*
4	Y, correct	Y	N	Y, correct	Y, correct	*Y, incorrect*	*Y, incorrect*
5	Y, correct	Y	N	Y, correct	Y, correct	N, correct	N, correct

6	Y, correct	Y	N	Y, correct	Y, correct	N, correct	N, correct
7	*Y, incorrect*	N		*Y, incorrect*	N, correct	-	-
8	Y, correct	Y	N	Y, correct	Y, correct	*Y, incorrect*	*Y, incorrect*
9	*Y, incorrect*	N		*uncertain*	*uncertain*	-	-
10	*Y, incorrect*	N		N, correct	*Y, incorrect*	-	-
11	Y, correct	Y	Y	Y, correct	Y, correct	Y, correct	Y, correct
12	Y, correct	Y	Y	Y, correct	Y, correct	Y, correct	Y, correct
13	Y, correct	Y	N	Y, correct	Y, correct	N, correct	N, correct
14	Y, correct	Y	N	Y, correct	Y, correct	N, correct	*uncertain*
15	Y, correct	Y	Y	Y, correct	Y, correct	Y, correct	Y, correct

**CSC = covered stent correction, Int = interventionist, PVP = pulmonary vein protection, VR = virtual reality*
